# The emerging dental workforce: why dentistry? A quantitative study of final year dental students' views on their professional career

**DOI:** 10.1186/1472-6831-7-7

**Published:** 2007-06-15

**Authors:** Jennifer E Gallagher, Resmi Patel, Nora Donaldson, Nairn HF Wilson

**Affiliations:** 1 King's College London Dental Institute At Guy's, King's College and St Thomas' Hospitals, Department of Oral Health Services Research & Dental Public Health, Oral Health Workforce & Education Research Group, London, UK; 2 King's College London Dental Institute At Guy's, King's College and St Thomas' Hospitals, Office of the Dean and Head of School, Oral Health Workforce & Education Research Group, London, UK

## Abstract

**Background:**

Dental graduates are joining a profession experiencing changes in systems of care, funding and skill mix. Research into the motivation and expectations of the emerging workforce is vital to inform professional and policy decisions. The objective of this research was to investigate final year dental students' perceived motivation for their choice of career in relation to sex, ethnicity and mode of entry.

**Methods:**

Self-administered questionnaire survey of all final year dental students at King's College London. Data were entered into SPSS; statistical analysis included Chi Squared tests for linear association, multiple regression, factor analysis and logistic regression.

**Results:**

A response of 90% (n = 126) was achieved. The majority were aged 23 years (59%), female (58%) and Asian (70%). One in 10 were mature students. Eighty per cent identified 11 or more 'important' or 'very important' influences, the most common of which were related to features of the job: 'regular working hours' (91%), 'degree leading to recognised job' (90%) and 'job security' (90%). There were significant differences in important influences by sex (males > females: 'able to run own business'; females > males: 'a desire to work with people'), ethnic group (Asians > white: 'wish to provide public service', 'influence of friends', 'desire to work in healthcare', having 'tried an alternative career/course' and 'work experience') and mode of entry (mature > early entry: 'a desire to work with people'). Multivariate analysis suggested 61% of the variation in influences is explained by five factors: the 'professional job' (31%), 'healthcare-people' (11%), 'academic-scientific' (8%), 'careers-advising' (6%), and 'family/friends' (6%). The single major influence on choice of career was a 'desire to work with people'; Indian students were twice as likely to report this as white or other ethnic groups.

**Conclusion:**

Final year dental students report a wide range of important influences on their choice of dentistry, with variation by sex, ethnicity and mode of entry in relation to individual influences. Features of the 'professional job', followed by 'healthcare and people' were the most important underlying factors influencing choice of career.

## Background

### Challenges facing health professions

Societal, political and economic change impacts on the healthcare workforce, which is recognised as 'critical' for health systems. [1,2] Furthermore, the skills, motivation and commitment of the health workforce in general are increasingly recognised as being intimately linked with the performance of health systems, and thus important areas for research. [3] Sociologists' suggest that professionals are motivated by status in the financial and social orders, [4,5] and that professional groups are facing challenges in their relationships with governments across Europe, [6] and the breaking down of traditional demarcations between professional groups. [7] In addition, there is some evidence of a generational effect in the workplace, with the suggestion that the emerging workforce has very different professional expectations than those in positions of authority and leadership, who are generally several decades older. [8,9] Johnson et al., [6] suggest that the reaction and ability to address these challenges, which often emanate from forces outside of the immediate professional field, is critical to the future nature of professions. Research into the motivation and professional expectations of the emerging workforce is therefore vital to provide evidence to inform professional leadership and policy decisions. Within dentistry, such research is recognised to be especially important given the length and cost of training; [10] however, research in this field is limited and may not take account of the contemporary student intake. [11]

### Dental workforce facing change

New graduates from UK dental schools are emerging into a profession facing a time of unprecedented change, both general as outlined above, and specific to the profession. [12] Fundamental changes to state funded dental care, include the introduction of a new dental contract for National Health Service [NHS] dentistry in England and Wales, with limits to the volume of state care that may be provided, [13-15] a significant shift of dental care to the private sector, [16] pressure on jobs from international graduates, [17] increased emphasis on skill-mix development within the dental team; [18] professionalisation of dental care professionals [DCPs]; [19] and continual innovation in dental techniques, products and materials [20]. New graduates face a professional career, which will be characterised by change in the 21^st ^century. [21]

### Changing nature of the profession

As with entrants to university in general and other professions, such as medicine, admissions to dentistry include an increasing proportion of females and ethnic diversity. [22-25] Dentistry has been particularly effective in attracting minority ethnic groups during the 1990's as demonstrated by Bedi and Gilthorpe; [22] and evidenced by UCAS admissions statistics. [24] As with medicine, [26-29] there are concerns that the feminisation of the profession will result in greater workforce challenges and this is reflected in recent workforce modelling exercises in England and Wales [30] and Scotland, [31] together with recommendations for systems reform to facilitate their contribution to the professional workforce. [32] Recent concerns over workforce shortages in certain parts of the country have led to formal recruitment exercises in England, both international and nationally. [17,33] Together with natural workforce immigration, this has resulted in more dentists from abroad joining the Dentists Register in 2005 than home students, [19] and more of the new NHS recruits coming from outside the UK. [34] In addition to the above, recent expansion of dental student numbers from September 2005 onwards announced by the Secretary of State for Health, [35] has provided additional opportunities for more young people, including increasing numbers of graduates, to pursue a career in dentistry. [24]

### Educational changes

Changes in education policy have resulted in students emerging with significant levels of student debt. [36] New entrants will now face the additional challenge of top-up-fees; [37] these are likely to impact on admissions and result in financially driven long term professional decisions. It has been suggested that dental students are more likely to be affected as they have a longer academic career and shorter holidays than other students, with the result that their may be implications for their future location and system of working, even to the extent that new graduates may be less likely to wish to work amongst the more disadvantaged sections of our community. [38] Nonetheless applications for graduate entry programmes in dentistry are heavily oversubscribed.

### What is known already about choice of dentistry as a career?

The motivation for studying dentistry has come under scrutiny amongst applicants to, and students of various schools across the UK, [10,39-41] and beyond. [42-48] This has included comparisons with medical counterparts. [40] Past studies within King's College London Dental Institute have revealed that applicants appear to have a more idealised view of the profession than final year students; [10] however, both cohorts were largely concerned by professional and personal matters. Comparison of medical and dental students across six dimensions of interest (status and security, nature of the occupation, career opportunities, patient care/working with people, use of personal skills and interest in science) by Crossley and Mubarik, [40] suggested that dental students were significantly more likely to be motivated by 'status and security', 'high income' and the 'nature of the occupation', when compared with their medical counterparts; whereas medical students were significantly more likely to be motivated by 'altruism' than dental students. However, the lack of altruism identified in their sample of students is not supported by other studies, [10,41,48,49] and researchers in the field. [50] None of the above studies has examined motivation in relation to all three demographic variables of sex, ethnicity and admission type, which given the changing nature of the profession is an important area for research.

### King's College London Dental Institute (KCLDI)

KCLDI is the largest dental school in the UK and amongst the largest in Europe. The Institute contributes up to 25% of the home trained dentists to the profession in England, thus the views of its students, although not necessarily representative of the national picture, have a significant impact on the profession. UCAS data for 2003 and 2004 suggest that the dental school has a high proportion of students from ethnic groups relative to other schools, including medicine, within King's College London, [51,52] and that the College as a whole has a higher intake of minority ethnic groups than nationally. [24] As the majority of the schools' undergraduate students are female and from minority ethnic groups, researchers at KCLDI are therefore well placed to examine the motivation and views of new entrants to the profession.

### Programme of research

The findings presented in this paper represent part of a larger programme of research the aim of which is to investigate the views of final year dental students', and recent graduates, on their future professional careers and explore how they consider their working lives may be enhanced. Both qualitative and quantitative methodological approaches have been utilised, with the qualitative data informing the development of a questionnaire instrument for the quantitative research as well as providing the theory underpinning student views derived from qualitative analysis.

This paper reports the reasons that final year students at the end of their course perceive have influenced their choice of dentistry as a professional career and relates them to the current educational and health policy. Subsequent papers will report on students short- and long-term future plans in more detail and the theory emerging from the qualitative data.

The objective of the research presented in this paper was to investigate final year dental students' perceived motivation for their choice of dentistry as a professional career in relation to sex, ethnicity and type of entry to dental school.

## Methods

The questionnaire instrument was constructed using qualitative data from the focus groups of final year dental students at KCLDI and vocational dental practitioners nationally during 2004/05 and drawing on the published literature. The study was undertaken using the approach outlined by Dillman. [53] King's College London Ethics Committee approved the protocol for this study of final year dental students and the questionnaire instrument (KCLREC: 03/04-109). The questionnaire was piloted on a representative sample of students who were not part of the study population.

The final version of the questionnaire had a total of 27 questions, 11 of which contained subdivisions. The instrument comprised five sections examining their vision of dentistry (i.e. why they had chosen it as a career), short-term career aspirations, long-term career aspirations, influences on their career and their personal details.

The class list of all final year dental students in 2004/05 was requested from the School Registry and all students (n = 140) were included as the study population, 57% of whom were female and 70% Asian.

To obtain maximum response and minimise disruption to academic studies, the questionnaires were distributed following the Fifth Year (Final) examinations in mid-June. All students were offered the opportunity to complete a questionnaire. This survey was conducted anonymously. The tailored approach to the conduct of a questionnaire survey recommended by Dillman, [53] was used to maximise the response rate. An e-mail informed the students that they would be receiving a request to help with this study. Students then received the questionnaire with a covering letter via the Registry. A reminder was sent after three weeks and replacement questionnaire with follow-up letter in six weeks. Finally after eight weeks students received a final reminder/thank you letter informing them that the survey was closing. Students were informed that completion and return of the questionnaire implied consent to participate in the study. This questionnaire took approximately 15 minutes to complete. Data were entered onto computer and analysed using the Statistical Package for Social Sciences (SPSS) Version 14.0 for Windows.

Descriptive analysis was undertaken to present an overview of the findings from this population study, together with an analysis by sex, ethnicity, and mode of entry (direct or mature entry) to study. Differences between groups were examined using Chi-squared test for linear trends across the rated questions.

In examining their 'vision' of dentistry, students were asked to identify how much they considered each of the 23 factors had influenced their choice of dentistry. The 'vision' section of the questionnaire explored why students perceived they had chosen dentistry as their professional career. Closed questions were utilised permitting students to scale responses such as why they chose dentistry, from "very important" (Score – 1) to "not important" (Score – 5) on a five-point Likert scale across 23 items. This section also covered questions to explore students' perception on alternative careers, in particular medicine and reasons for not pursuing alternative career pathways. Demographic details obtained included age, sex, and ethnicity, year of entry, mode of entry, qualifications and the level of existing debts.

Proportions of respondents favouring each of the factors are provided with the corresponding 95% confidence intervals. Similarly, the distribution of the major influencing factors quoted is provided. A factor analysis of this question was used to determine the principal latent determinants of the choice of dentistry as a career, and aggregate scores were derived for each principal determinant in order to rank their impact and find the most important independent driving force on this choice.

Linear regression models were then used to assess the relationship of demographic variables and their proposed plans for the future with the principal determinants and factors perceived as influencing their future careers. Finally, the corresponding relationships with the modal category of the single most important influence on their choice of dentistry were investigated using a logistic regression model.

## Results

The results are in four sections: first, the response to the study; second univariate analysis; and third, multivariate analysis of the factors which students' perceive influenced their choice of dentistry as a career; fourth and finally, other career related issues are explored.

### Response

Of the 140 questionnaires distributed to the Year 5 dental students, 126 were completed and returned giving an overall response rate of 90%. The age of respondents ranged from 22–33 years, with a mode of 23 years (59%). Thirteen respondents (10.5%) were mature students, aged 26 years and over.

Females (58%) exceeded males (42%). The majority of the respondents identified their ethnicity as Asian (70%) followed by White (22%) (Table 1). The majority of Asian respondents reported being Indian, representing 54% of total respondents and thus the single largest ethnic group. Two out of four students were Asian females (n = 52; 41%).

**Table 1:** Sex and ethnicity of final year dental student respondents (n = 126)

**Sex**		**ETHNIC GROUP**							**Total**
			**Asian**						
		**White**	**Asian: Indian**	**Asian: Pakistani**	**Asian: Bangladeshi**	**Asian: Other**	**Asian Total**	**Other**	**All**

**Male**	No.	12	29	2	1	4	36	5	53
	*% of Total*	*9.5%*	*23%*	*2%*	*1%*	*3%*	*28.5%*	*4%*	*42%*
**Female**	No.	16	39	0	2	11	52	5	73
	*% of Total*	*13%*	*31%*	-	*2%*	*9%*	*41%*	*4%*	*58%*
**Total**	**No.**	**28**	**68**	**2**	**3**	**15**	**88**	**10**	**126**
***Total***	***% of Total***	***22%***	***54%***	***2%***	***2%***	***12%***	***70%***	***8%***	***100%***

The majority of students (76%) identified that they had debts, 45% of whom had debts of £21,000 or more. Students reporting debt did not differ markedly from the overall profile of respondents in terms of age, sex or ethnicity.

### Influences on choice of dentistry as a professional career: univariate analysis

#### Important influences: multiple response

**Figure 1:**
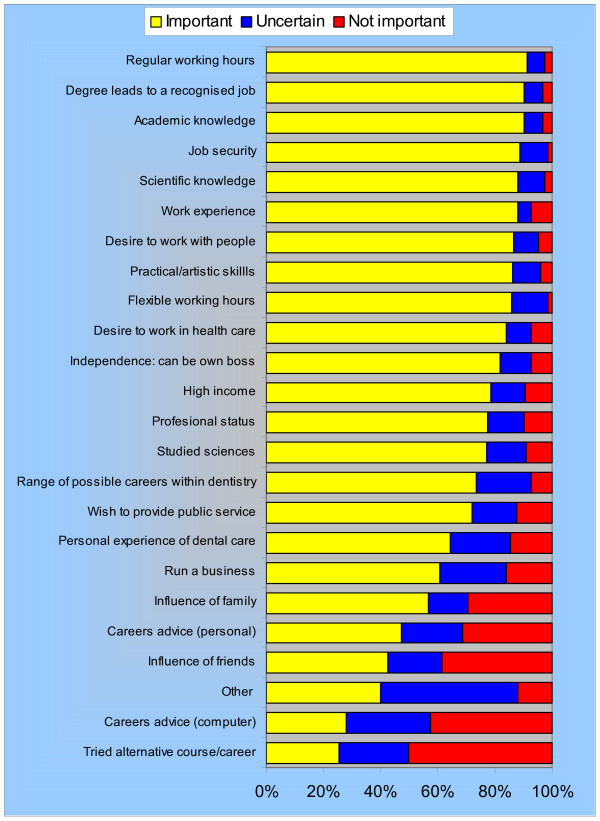
Factors influencing students' choice of dentistry: multiple responses (n = 126).

Students were asked to score their level of support for a wide range of influences according to the level of importance they perceived the factor had in their choice of a professional career in dentistry (Figure 1). Eighty per cent identified 11 or more influences as being 'important' or 'very important' with over half the students indicating that 17 or more influences were 'important' or 'very important' in their choice of dentistry as a professional career. Combining 'important' and 'very important' influences, the top five were: 'regular working hours' (91%), 'degree leading to recognised job' (90%), 'job security' (90%), 'academic knowledge' (90%), and 'scientific knowledge' (88%). In contrast, 'careers advice' and the influence of 'family' and 'friends' were the least commonly acknowledged influences on respondents' choice of dentistry.

**Figure 2:**
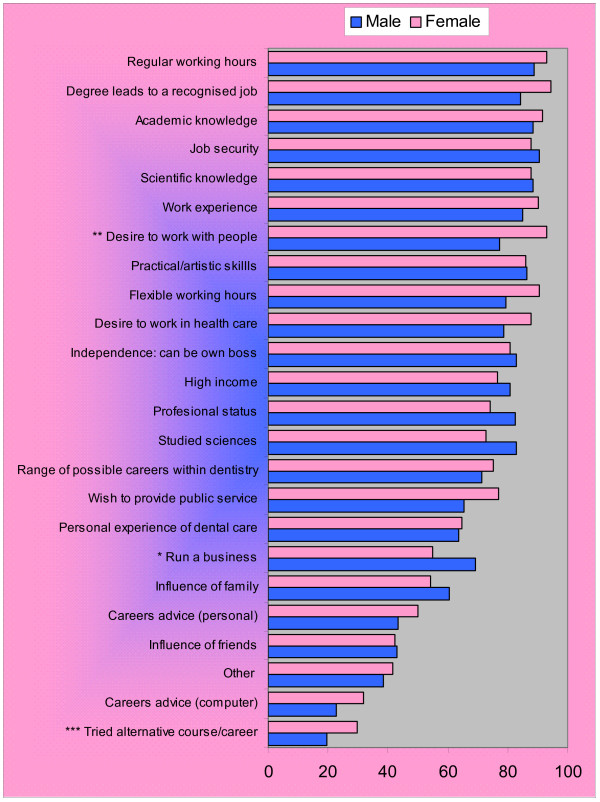
**Important and very important factors influencing students' choice of dentistry by sex: multiple responses (n = 126)**. Note * males > females, p = 0.026. ** females > males, p = 0.028. *** females > males, p = 0.028.

**Figure 3:**
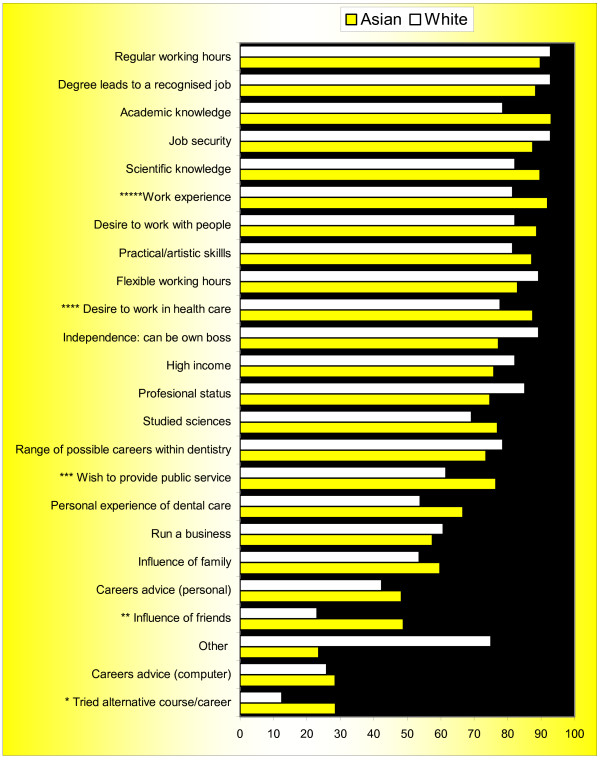
**Important and very important factors influencing students' choice of dentistry by ethnicity: multiple responses (n = 116)**. **Note **1. Asians were significantly more likely to identify the following influences: * tried an alternative career/course, p = 0.004. ** been influenced by friends, p = 0.007. *** wish to provide public service, p = 0.005. **** have a desire to work in healthcare, p = 0.027. ***** been influenced by work experience, p = 0.038. 2. Influence of family (p = 0.057) and academic knowledge (p = 0.07) approaching significance

There was variation by age, sex and maturity on entry, in relation to the influences ranked as important, with three of the top five influences in common (Figures 2, 3).

'Job security' was the top influence of importance for males (91%), followed by 'regular working hours' (90%), whereas for females 'degree leading to recognised job' (95%) and a 'desire to work with people' (93%) were most important (Figure 2). Significantly more males (69%) than females (55%) perceived that being able to 'run a business' (p = 0.03) was important in influencing their choice of dentistry, whereas a 'desire to work with people' (p = 0.03) was a significantly higher influence on career choice for females.

Asian students identified ' academic knowledge' (93%), 'work experience' (91%) and 'regular working hours' (91%) most frequently as influences of importance, whereas white students identified 'degree leading to recognised job' (93%) and 'regular working hours' (93%) (Figure 3). Significantly more Asian than white students reported having 'tried an alternative career/course' (p = 0.004), a 'wish to provide public service' (p = 0.005), the 'influence of friends' (p = 0.007), a 'desire to work in healthcare' (p = 0.03) and 'work experience' (p = 0.04) as being of importance.

Direct entrants were significantly more likely to perceive 'work experience' (p = 0.027) as important in choosing dentistry, than mature students (88% cf 77%).

#### Very important influences: multiple response

The top five 'very important' influences for all respondents displayed in Table 2. They are predominately job related: 'job security' (57%); degree leading to recognised job (49%) independence (48%) and regular working hours (44%) but include a desire to work with people (50%). Amongst male respondents, the top 'very important' influence was 'job security' (66%), whereas for females it was a 'desire to work with people' (53%). Job security was also top or joint top for white and Asian students respectively. Two of the top three 'very important' influencing factors for mature students were different from the other sub-groups: regular working hours (61%) and range of possible careers (54%).

**Table 2:** Top five 'very important' influences in student population overall and by sub-group: multiple responses

	**Very Important Influences**				
**Group**	**1^st^**	**2^nd^**	**3^rd^**	**4^th^**	**5^th^**
**All**	Job security 57%	Desire to work with people 50%	Degree leading to recognised job 49%	Independence 48%	Flexible working hours 46%

**Sex**					
Males	Job security 66%	Independence 55%	Degree leads to recognised job 49%	Practical skills 47%	Desire to work with people *and *Desire to work in healthcare 45%
Females	Desire to work with people 53%	Desire to work in health care 52%	Flexible working hours 47%	Independence 44%	Wish to provide a public service 40%

**Ethnicity**					
Asian	Job security 56%	Desire to work in healthcare 53%	Desire to work with people 51%	Independence 45.5%	Flexible working hours 45.5%
White	Job security 54% Degree leading to a recognized job 54%		Desire to work with people 50% Independence 50% Work experience 50%		

**Time of entry**					
Mature students	Regular working hours 61%	Independence 54% Range of possible careers 54%		Degree leading to recognised job 46% Tried alternative course/career 46% Flexible working hours 46% Job security 46% High income 46% Desire to work in health care 46%	

direct entry students similar to overall picture

### Major influence on choice of dentistry as a professional career: univariate analysis

#### Major influence: single response

**Figure 4:**
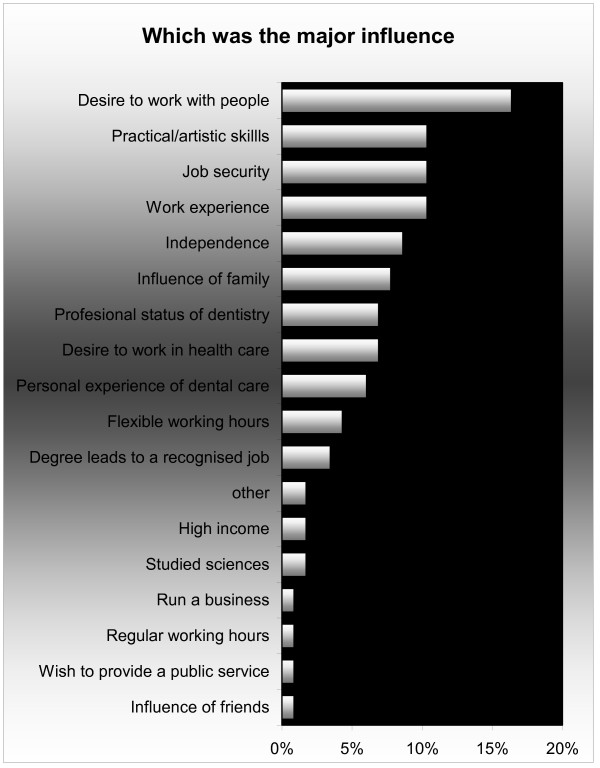
Major influence on choice of dentistry as career: all respondents (n = 116).

Having identified to what degree the full range of influences had influenced their choice of dentistry, students were asked to specifically identify the single *major *influence on their career of choice. Only 17 of the same 23 listed options were utilised by respondents (Figure 4). Overall the single major influence was identified as a 'desire to work with people' (16%), followed by 'work experience' (10%), 'job security' (10%), 'independence' (8%), 'influence of family' (7%) and 'professional status of dentistry' (6%). The small group of respondents (7%) who identified 'family influence' as the major influence was almost exclusively female (90%) and from minority ethnic groups (90%).

**Figure 5:**
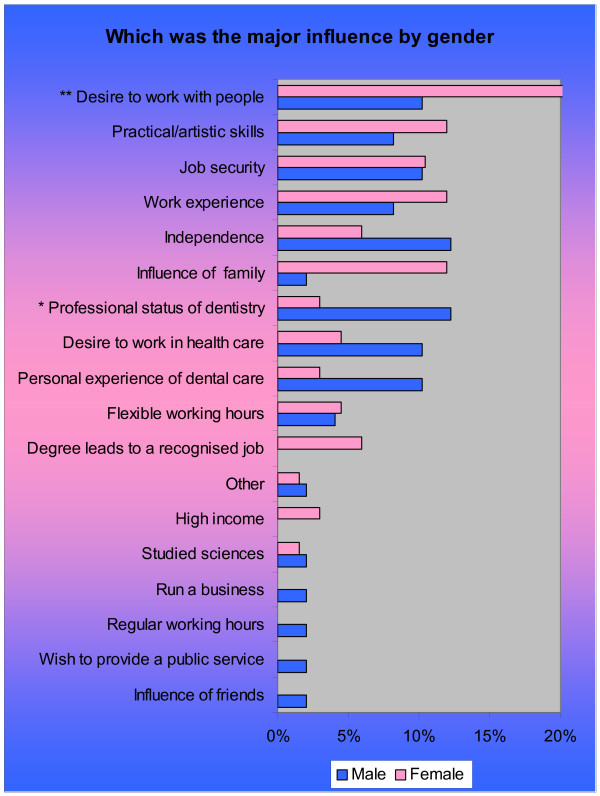
**Major influence on choice of dentistry as career: by ethnicity (n = 107)**. Note * males > females, p = 0.05. ** females > males, p = 0.03.

The single major influence of the majority of male respondents was 'professional status of dentistry' (19%, n = 6) (Figure 5). 'Significantly more males than females identified that their choice of career was influenced by the 'professional status of dentistry' (p = 0.05). Among the 73 female respondents, the single major influence in choosing dentistry was a 'desire to work with people' (19%) and they were significantly more likely than males to express desire to work with people as the major influence (p = 0.03).

**Figure 6:**
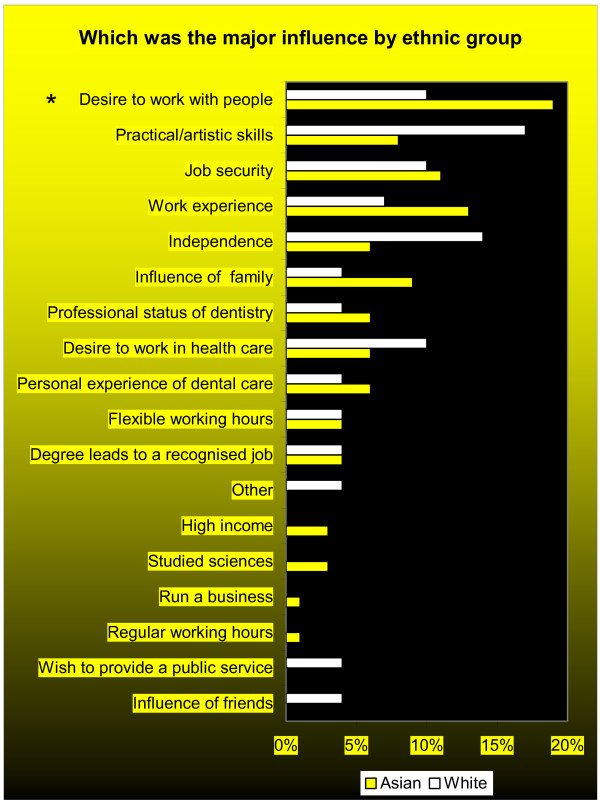
**Major influence on choice of dentistry as career: by ethnicity (n = 107)**. **Note**: * Asians > white students, p = 0.03.

The major influence reported by Asian students was also 'desire to work with people' (18%). Asian students were significantly more likely than white students to consider 'desire to work with people' (p = 0.03) as their major influence in choosing their professional career. In contrast, white respondents considered 'practical/artistic skills' (18%) as their major influence.

The major influence for mature students was 'practical/artistic skills' (23%, n = 3); however, significantly more direct entrants than mature students considered a 'desire to work with people' (p = 0.03) as the major influence on career choice.

### Influences on choice of dentistry as a professional career: multivariate analysis

#### Single most important influence: logistic regression

A logistic regression model showed that *ethnicity *was the only significant factor influencing the modal choice for the single most important influence on choice of dentistry as a career, i.e. a 'desire to work with people'. The odds of the 'desire to help people' being the driving force in choosing dentistry as a career were more than double for the Indian group, in relation to white, Pakistani, Bangladeshi and Other-Asian groups (OR = 2.4; 95% CI 1.1 to 5.3; p = 0.04).

#### Principal latent determinants of career choice: factor analysis

The results of the maximum likelihood factor analysis using varimax rotation are set out in Table 3. Five principal factors explained 61% of the total variability contained in the 23-item question relating to their vision of dentistry as a career. The first factor, explaining 31% of the variability, was related to professional status, job security and independence. This will be referred to as the ***professional job factor***. The second factor, which we will refer to as the ***healthcare-people factor***, explained 11% of the variability and was comprised of items related to the desire to work with people, in healthcare or public service. The third factor was the ***academic-scientific factor ***and explained 8% of the total variability. The fourth and fifth factors, each of which explained 6% of the variability, referred to influences from ***careers advisers ***and ***family and friends***.

**Table 3:** Factor Analysis: influences on choice of dentistry reported by final year dental students

	**Factor 1**	**Factor 2**	**Factor 3**	**Factor 4**	**Factor 5**
	**31%**	**11%**	**8%**	**6%**	**6%**
	**Professional job**	**Healthcare and people**	**Academic**	**Careers advising**	**Family and Friends**

personal experience of dental care	0.26	0.03	0.04	0.27	0.28
work experience	0.14	0.21	0.01	0.28	-0.04
careers advice (computer)	0.06	0.06	0.13	0.79	0.14
careers advice (personal)	0.04	0.15	0.06	0.87	0.23
having studied sciences	0.15	0.23	0.25	0.21	-0.03
having tried alternative	-0.13	0.05	0.03	0.34	0.26
influence of family	0.12	0.00	-0.02	0.12	0.79
influence of friends	0.06	0.22	0.07	0.24	0.72
desire to work with people	0.21	0.57	0.20	0.09	0.29
desire to work in health care	0.14	0.65	0.27	0.11	0.03
wish to provide public service	0.08	0.70	0.08	0.19	0.13
professional status of dentistry	0.53	0.29	0.18	0.06	0.31
high income	0.49	0.24	0.18	0.10	0.31
regular working hours	0.64	0.29	0.29	-0.10	0.09
flexible working hours	0.48	0.37	0.25	0.05	0.11
independence	0.84	0.08	0.11	0.10	-0.03
being able to run a business	0.78	-0.07	0.04	0.08	0.01
job security	0.58	0.42	0.25	0.09	0.01
academic knowledge	0.23	0.17	0.93	0.10	0.15
scientific knowledge	0.28	0.32	0.81	0.09	0.01
practical skills	0.28	0.39	0.40	0.17	-0.19
degree leading to recognised job	0.33	0.31	0.19	0.09	0.06
range of possible careers	0.09	0.14	0.13	0.02	0.10

Table 4 shows the multiple linear regressions for these five factors in terms of the demographic and other variables. *Living standards *in future career choices were considered the main influential determinant for the ***professional job factor***, followed to a lesser extent by sex. The students that scored high on this factor tended to be those who attach high importance to the standard of living in making future professional choices (coefficient = 0.43; 95% CI 0.14 to 0.71; p = 0.004) and male (coefficient = 0.28; 95% CI -0.04 to 0.60; p = 0.09). The model was adjusted for age (p = 0.22) and ethnicity (p = 0.53), although neither of these variables was shown to have an influential effect on the job factor.

**Table 4:** Multiple linear regression models for the five principal latent factors that drive the influences for final year dental students

**Factor I: PROFESSIONAL JOB FACTOR**						
**Coefficients^a^**						

		Unstandardized Coefficients			95% Confidence Interval for B	
					
Model		B		Sig.	Lower Bound	Upper Bound

1	(Constant)	-3.046		.081	-6.476	.383
	Sex	.277		.089	-.043	.598
	Age	.078		.287	-.066	.222
	Indian	.195		.245	-.135	.526
	Standard of living important in future prof. choices	.425		.004	.143	.708

a. Dependent Variable: Job						

**Factor II: HEALTHCARE-PEOPLE FACTOR**						

**Coefficients^a^**						

		Unstandardized Coefficients			95% Confidence Interval for B	
					
Model		B	t	Sig.	Lower Bound	Upper Bound

1	(Constant)	1.266	.809	.421	-1.840	4.372
	Age	-.065	-.965	.337	-.198	.068
	Sex	-.197	-1.200	.233	-.524	.129
	Indian	-.299	-1.759	.082	-.636	.038
	Importance of family commitments in no. long term sessions	.336	3.800	.000	.161	.511

a. Dependent Variable: Altruism						

**Factor III: ACADEMIC-SCIENTIFIC FACTOR**						

**Coefficients^a^**						

		Unstandardized Coefficients			95% Confidence Interval for B	
					
Model		B		Sig.	Lower Bound	Upper Bound

1	(Constant)	-1.492		.417	-5.123	2.140
	Age	.047		.540	-.105	.199
	Sex	-.066		.735	-.454	.322
	Indian	.017		.934	-.389	.423
	Team work important in future prof. choices	.218		.067	-.015	.452

a. Dependent Variable: Academic						

**Factor IV: CAREERS-ADVISING FACTOR**						

**Coefficients^a^**						

		Unstandardized Coefficients			95% Confidence Interval for B	
					
Model		B		Sig.	Lower Bound	Upper Bound

1	(Constant)	-3.200		.059	-6.517	.118
	Age	.120		.078	-.014	.255
	Sex	-.001		.998	-.348	.347
	Indian	-.029		.876	-.391	.334
	Important of career opportunities re where work in short term	.189		.058	-.006	.383

a. Dependent Variable: Careers Advising						

**Factor IV: FAMILY-FRIENDS-INFLUENCE FACTOR**						

**Coefficients^a^**						

		Unstandardized Coefficients			95% Confidence Interval for B	
					
Model		B		Sig.	Lower Bound	Upper Bound

1	(Constant)	1.444		.374	-1.761	4.649
	Age	-.050		.450	-.182	.081
	Sex	-.130		.450	-.470	.210
	Indian	-.134		.457	-.488	.221

a. Dependent Variable: Family-Friends						

The students that scored high on the ***healthcare-people factor ***tended to be those who gave more importance to *family commitments *in their future professional choices (coefficient = 0.33; 95% CI 0.11 to 0.55; p = 0.004). Neither age, nor sex, nor ethnicity showed an association with the healthcare-people factor.

The students that score high on the ***academic-scientific factor ***tended to be those who attached more importance to *team work *when making decisions about their professional career in the short-term decisions (coefficient = 0.22; 95% CI -0.02 to 0.45; p = 0.07). Neither age, nor sex, nor ethnicity showed any significant association with this factor.

The students that scored high on the ***careers-advising factor ***tended to be those who gave importance to career opportunities when considering short term plans (coefficient = 0.19; 95% CI -0.01 to 0.38). Although not statistically significant, to some extent they tended to be older (coefficient = 0.12; 95% CI -0.01 to 0.26; p = 0.08). Neither sex nor ethnicity demonstrated any association with this factor.

No variable showed a significant association with the factor ***family-friends-influencing factor***.

### Career choices and careers advice

As preliminary qualitative research identified that medicine had been an alternative career choice considered by many of the students, all students were asked whether they had considered choosing medicine as opposed to dentistry as their career of choice, together with reasons for not pursuing a career in medicine. Almost half of the respondents (n = 61; 48.5%) and 55% of males had considered medicine as an alternative career. There was no difference between the two main ethnic groups.

**Figure 7:**
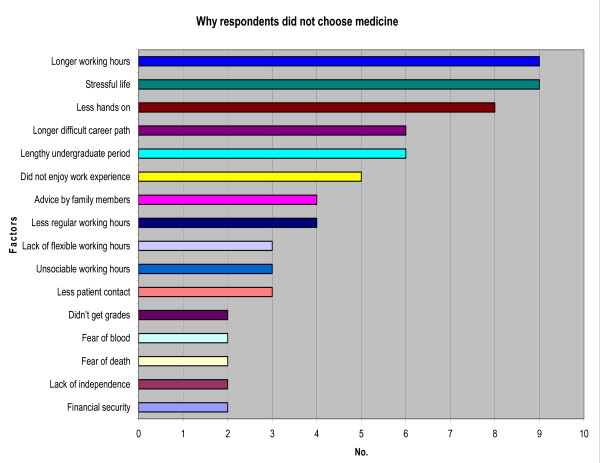
Reasons for rejection of medicine as an alternative career by final year dental students (48%; n = 61).

Medicine was actively rejected as a career option by dental students, for a range of reasons reported in open response (Figure 7). These included: 'longer working hours' (18%); 'stressful life' (18%); 'less hands-on' approach to care (16%), 'lengthy undergraduate period' (12%) and 'longer and difficult career pathway' (12%).

## Discussion

### Introduction

This section considers the contribution of the findings to the existing literature, examines the influences that have motivated students to choose dentistry as their professional career and the implications for health and education policy, professional action and future research.

### Contribution to the literature

This study contributes to the literature on dental workforce in three main areas. First, it examines the motives of final year students as they prepare to leave dental school, in contrast to the majority of published studies, which have focused on admissions or new entrants to dental school. [23,41,44-46,54] It could be argued that it is rather late in their studentship to explore this issue; however, there is evidence from two studies that the reported motivations of students were not significant across the academic years. [49] Furthermore, within the overall aim of the research programme, it provides the opportunity to relate these views to future career plans. Second, development of the research instrument was informed by qualitative research, which will be published separately, and thus it quantifies contemporary issues for the current profile of students. Most other studies have used instruments constructed for medical counterparts or based on existing literature. Third, it provides both univariate and multivariate analysis of the data from this population survey in relation to sex, ethnicity and type of entrant (direct vs mature), which makes an important contribution to any consideration of the changing demography of the dental student intake and therefore the future workforce.

The response rate amongst students was high (90%), thus providing support to generalise the findings to the final year population under study, particularly as non-respondents did not differ in terms of their demographic profile. Although the findings cannot be considered to be representative of dental students in general in the UK, the views of this group of students, given their demographic profile and the trends in admission to dental school, are nonetheless important to inform workforce planning and policy.

KCLDI has a profile that is skewed towards Asian students. [51,52] The majority are home students and will tend to be second or third generation in the UK, an issue which might be explored in future research. There is evidence from one UK dental school that Asian students are significantly more likely than their counterparts to have siblings or relatives (other than parents) in dentistry. [23] Dentistry's success in attracting Asian students is noteworthy; however, the fact that this is not matched across other ethnic groups within London is an area for further research, and action, as recommended by Stewart et al., [23] particularly given concerns that ethnicity of dentists may be related to the uptake of care by certain groups.

### Motivation

Building on the qualitative components of this research programme, this quantitative study has confirmed the wide range of influences on students' choice of career, lending support to the range of influences raised in similar studies of applicants to or students of dentistry. [10,23,40,44,50] Multivariate analysis provided additional insight into the basic dimensions or clusters of motives. There are some parallels in the groupings identified in the US study of first year dental students by Scarbecz and Ross, [44] those utilised by Crossley and Mubarik in the north of England, [40] Bernabe et al. in Peru, [45] and Vigild and Schwarz in Denmark. [48] Factor analysis was undertaken on the data from US and Danish students. Scarbecz and Ross identified eight factors, which accounted for 59% of the variability in first year students; [44] whilst Vigild and Schwarz identified eight factors representing 65% of variability in first year students. [48] In this UK study of final year students, just five factors provided for 61% of the variability. Whereas 'money' was highlighted as the prime factor in the US, followed by 'people', and then various features of the professional job, Danish students showed some variance over time with the most recent study in 1994 identifying 'people', followed by 'convenience/esteem' and 'work conditions' as the main factors. Amongst KCLDI students the ***professional job ***factor was dominant followed by ***healthcare-people***. Although the individual influence of 'high income' was an important influence for 78% of KCLDI students, it was not prominent in the factor analysis, when compared with US students, [44] and therefore similar to Danish students. [48] However, 'people' factors were secondary when compared with Danish students. [48] Each of the five clusters of motives from this study is examined in turn, starting with the leading factor.

#### Professional job factor

Professional job factors explained 31% of the variation with 'independence', 'being able to run a business', 'regular working hours' and job security' associated with the ***professional job related factor***. It is important to note that other aspects, close to being included were also job related: 'high income', and 'flexible working hours'. Aspects of the professional job have scored highly in other studies amongst admitted and final year students at the same school, [10] and beyond. [40,44,48,49] The primary reasons for choosing dentistry amongst Irish students were related to features of the professional job, [49] and dental students in Manchester reported being motivated by dimensions of 'status and security' followed by the 'nature of the occupation'; [40] which together also relate to aspects of the ***professional job***.

Multivariate analysis suggested that students attaching high importance to this factor also attached importance to future living standards and were more likely to be male. This supports sociologists perspectives on professions that their members are motivated by status in the social and economic orders, [4,5] and past surveys of applicants to, and students of, dentistry. Comparing the factor analysis with the univariate analysis of the data, the findings suggest that different aspects of the ***professional job ***may be important to sub-groups with males showing significantly more interested in 'professional status' and 'business', whereas females were more interested in 'independence' and 'flexibility of working hours', as demonstrated in past research. [23,40,48,49] However, past sociological views of professions will have been informed by male-dominated professions, which may explain why professional status was significantly more likely to be identified by males as the single major motivating factor in choosing dentistry, when compared with females. Given the changing demography of the student population, this may alter over time. There appears to be less interest amongst KCLDI students in the business aspects of dentistry than US counterparts. [44]

Work experience, which provides insight into features of the professional job, was reported as an important influence for 87% of students. Looking to the future, it is clear that the features of the professional role of dentists and their day-to-day job are likely to change significantly to the extent that it will be rather different to that encountered by current cohorts on work experience. There is anticipated to be much greater emphasis on 'team-working' rather than 'independence', [18] and increased freedom for corporate bodies in the running of primary dental care, [55] both of which are likely to lead to larger practices, with a resultant reduction in the volume of business opportunities than in the past. There is also the need to better facilitate career satisfaction within the dental team. [56] Patient demands for longer opening hours in primary care may challenge the 'regular working hours' of traditional dental practices. [57] There are now more entrants to the professional register from abroad, [58] and more dentists in training. [35] Traditional job security appears to be threatened by these new ways of working, together with pressure on limited NHS resources. [59] Furthermore, there is some concern that the ability of females to work flexibly may be limited by the current contracting requirements of the new dental contract. [60]

If the influences that attracted dental students to their chosen professional career are being, or will be, challenged over the forthcoming decades, it is important that as a dental profession we provide a realistic view to those who seek work experience and apply to dental school. Skelly and Flemming have already stressed the importance that those advising and recruiting people into the profession should ascertain whether applicants have a realistic idea of what lies ahead. [10] Potential applicants need to be aware of how dentistry as a professional career may change over time in relation to skill-mix, team-working, techniques and materials, highlighting that the ability to respond flexibly and to address the various challenges can result in a stimulating career. Along with effective mentoring, identified by Moats-Kennedy as particularly appropriate for this generational cohort, [8,61] this will be important to minimise disillusionment with professional life and facilitate a positive approach to change. [8,61,62] However, professional and policy leaders also have a responsibility to recognise and minimise barriers that will negatively and inappropriately influence the ability of young dentists to contribute to the workforce.

#### Healthcare-people factor

People and healthcare issues were the second underlying factor, suggesting an element of altruism but not as strong as with Danish students in 1972 and 1994. [48] Whereas there was no age, sex or ethnic association with the factor as a whole, these variables were associated with individual components of the factor. A 'desire to work with people' was the second 'very important' factor in the choice of dentistry amongst all students (50%) and the most important for females (53%). This desire to work with or help people is reflected in the findings of most equivalent studies either as a primary or secondary influence, [23,44,45,48,49] and there is a growing body of evidence that suggests it is more common in females, [44,45] and that altruistic motivations may be secondary to positive perception of working conditions. [49] The finding that the odds of Indian students choosing a 'desire to work with people' being more than double that for white or other ethnic groups is an important finding and one which should be explored in future research.

The other aspect of this factor, 'a desire to work in healthcare' was the second very important influence for females (52%) and Asians (53%). Factor analysis suggested that the ***healthcare-people factor ***was significantly related to 'family commitments' and this has implications for policy makers, managers and business owners as it will be important to facilitate commitment to family for these people-orientated dentists who can make an important contribution to professional care in terms of valuing patient contact and form a significant proportion of the profession. In addition to the professional job changing, the systems of providing healthcare are changing with increasing privatisation of dental care, [16,63] and possibly over time greater emphasis on the use of state resources for priority groups. This is altering the nature of public service provided by dentists as it moves from being equivalent to medicine towards other professions such as law within the UK and dentistry.

#### Academic-scientific factor

Dentistry provides a clear avenue for pupils from a scientific background wishing to pursue an academic career. Other studies have identified a recurring 'interest in sciences' and the 'scientific nature of dentistry'. [23,48] Both these factors are generic to healthcare professions and will equally apply to medicine as to dentistry, with students being drawn from the same pool. Univariate analysis suggested that the academic nature of dentistry was one of the top important influences on the choice of dentistry by Asians, who clearly value this aspect of the profession. Although medicine has been recognised as one of the most popular alternatives for dental students, [23,44,48,49] and in the past some students have entered dentistry because they did not achieve the grades for medicine, the situation appears to be changing. The entry grades for dentistry are now as high, and in some institutions, including KCLDI, higher than medicine. That being the case, there is theoretically a higher academic status associated with entering dentistry. The declining volume of students studying scientific subjects in secondary school is a matter of public concern and therefore dentistry may be competing with other medical courses for a reducing pool of students in future.

#### Careers-advising factor

Faced with an ever expanding range of programmes available for study at university, assistance with choice becomes ever more important. In this technological age, computer-based advice through decision aid programmes was important for just over one quarter of students, with personal advice still more important for almost half of the total. Evidence from the multivariate analysis that those who report careers advice influenced their choice of dentistry are more likely to be influenced by the range of career opportunities in dentistry is important to note. Whether this relates to the quality of careers advice or the personality of the student seeking career advice, to inform a rational approach to decision making, is unknown; however these students' tended to be older and therefore perhaps especially anxious to have clear career plans. There are now more graduate entry students who will be older on exit from dental school; therefore these findings suggest that there may be more demands for careers advice within dentistry from this group. Moreover, in this time of change in modernising dental careers, [64] changing entrance criteria to specialisation, [65] possible innovative new tiers of care, [66-71] it becomes more important to provide contemporary advice and support to students and new dentists on the future nature of dentistry, modes of working and the range of career options.

#### Family-friends factor

This factor involved only 6% of the variation and interestingly was not associated with age, sex or ethnicity. However, univariate analysis would suggest that 'family' were the important single major influence for a small group of individuals, who tended to be female and come from minority ethnic groups. These individuals only represent a minority of students and the data do not support generalisation to the study population.

### Implications: summary

This study has highlighted the underlying factors, which determine students' choice of dentistry as a professional career. The balance between professional and people-healthcare factors appears to differ across published research; however, in this group of students, professional job related factors are dominant. In considering the wider context of health and health services, it appears that elements of three factors, which together describe 50% of the underlying motivation, are under particular challenge: features of the professional job, healthcare-people, and academic-scientific factors. The findings raise important issues about health professionals' motivation and highlighted the need for entrants to the healthcare workforce to understand the dynamic nature of professions and the pace of change, along with the need for mentoring support in relation to career development. Longitudinal research into the workforce expectations and subsequent career decisions and pathways could contribute to professional debate and inform policy planning, as with our medical counterparts. [72,73] Given the rate and nature of change within health services, and the current emphasis on reshaping of the workforce, these are issues for healthcare in general.

## Conclusion

Final year dental students confirmed a wide range of influences as important or very important, in their choice of dentistry as a professional career. The underlying influencing factors relate to features of the 'professional job', followed by 'healthcare-people' factors and to a lesser extent 'academic-scientific', 'careers-advising' and family-friends'. There is evidence that students' sex, ethnicity and maturity on entry to dental school are associated with individual influences relating to these factors. As professional job factors are the dominant motivating influence amongst contemporary students, and most likely to change, this has implications for the motivation of this cohort of dentists.

## Competing interests

Three of the authors (JEG, ND and NHFW) are academic staff at King's College Dental Institute. NHFW is Dental Dean and Head of School.

## Authors' contributions

JEG and NHFW conceived and designed the overall research programme. JG led the development of the protocol, gained ethics committee approval, oversaw the fieldwork including development of the questionnaire, contributed to the data analysis, interpretation of results and led on writing of the paper. RP conducted the fieldwork, entered the data to SPSS and contributed to the descriptive analysis. ND undertook the multivariate analysis. JG, ND and NHFW contributed to the paper. All authors reviewed the final manuscript.

## Pre-publication history

The pre-publication history for this paper can be accessed here:


